# Supplementary material for: A Likelihood Approach for Real-Time Calibration of Stochastic Compartmental Epidemic Models
Source: PLoS Comput Biol. 2017 Jan 17;13(1):e1005257. doi: 10.1371/journal.pcbi.1005257 (PMC5240920; doi:10.1371/journal.pcbi.1005257)
Supplement: S1 File — (TAR.GZ) [file pcbi.1005257.s014.tar.gz › HSPH_Online-SI-Revision/EnKF07/n10-severe/n10-severe_EnKF2_table8.pdf]

1	newly 3 weeks spec	53.	347.25	{71., 761.}	568.208	0.777926	newly 3 weeks cumul	226.	2595.04	{706., 8807.}	1062.11	0.940309	newly 5 weeks spec	40.	118.47	{8., 214.}	240.575	0.541749	newly 5 weeks cumul	307.	2902.11	{790., 9905.}	858.779	0.880276	newly 43 weeks cumul	452.	3152.69	{955., 10 047.}	607.639	0.757635
2	newly 3 weeks spec	48.	107.85	{0., 52.}	253.854	1.31478	newly 3 weeks cumul	239.	674.72	{12., 485.}	272.008	0.599698	newly 5 weeks spec	25.	71.73	{0., 39.}	315.4	1.30252	newly 5 weeks cumul	302.	837.44	{17., 911.}	270.265	0.609224	newly 45 weeks cumul	375.	1064.98	{18., 3203.}	277.947	0.640782
3	newly 3 weeks spec	7.	121.23	{0., 449.}	1706.71	0.984746	newly 3 weeks cumul	64.	905.68	{92., 2799.}	1322.97	0.773125	newly 5 weeks spec	1.	36.54	{0., 157.}	3690.	1.09654	newly 5 weeks cumul	67.	1008.54	{92., 3233.}	1413.31	0.77367	newly 47 weeks cumul	68.	1056.16	{92., 3465.}	1461.26	0.774819
4	newly 3 weeks spec	37.	241.09	{6., 1026.}	585.541	0.684509	newly 3 weeks cumul	140.	1476.64	{40., 6134.}	984.686	0.842178	newly 5 weeks spec	17.	74.89	{2., 353.}	396.059	0.601083	newly 5 weeks cumul	186.	1683.9	{49., 7105.}	836.763	0.794781	newly 42 weeks cumul	207.	1785.3	{53., 7614.}	793.324	0.774737
5	newly 3 weeks spec	63.	19.38	{0., 69.}	90.6667	1.9981	newly 3 weeks cumul	261.	735.34	{193., 869.}	194.613	0.364218	newly 5 weeks spec	22.	3.5	{0., 9.}	96.6364	1.91015	newly 5 weeks cumul	329.	747.01	{207., 869.}	141.912	0.292241	newly 42 weeks cumul	383.	751.53	{223., 911.}	118.138	0.270263
6	newly 3 weeks spec	37.	269.99	{1., 1388.}	707.595	0.861346	newly 3 weeks cumul	120.	852.52	{12., 3588.}	631.983	0.608439	newly 5 weeks spec	13.	172.74	{0., 916.}	1331.69	1.2327	newly 5 weeks cumul	158.	1255.76	{13., 5743.}	726.709	0.625508	newly 43 weeks cumul	173.	1558.39	{13., 7288.}	836.133	0.650695
7	newly 3 weeks spec	17.	64.05	{0., 70.}	416.412	1.49836	newly 3 weeks cumul	136.	714.24	{60., 1070.}	457.029	0.388026	newly 5 weeks spec	12.	13.34	{0., 26.}	172.333	1.61504	newly 5 weeks cumul	163.	757.17	{62., 1070.}	405.025	0.385802	newly 42 weeks cumul	196.	770.02	{62., 1070.}	343.776	0.392123
8	newly 3 weeks spec	10.	100.94	{14., 210.}	910.6	0.882097	newly 3 weeks cumul	74.	1267.63	{457., 1585.}	1613.01	1.03002	newly 5 weeks spec	3.	23.48	{0., 51.}	710.667	0.848803	newly 5 weeks cumul	80.	1336.44	{464., 1726.}	1570.55	1.03409	newly 46 weeks cumul	83.	1365.43	{464., 1889.}	1545.1	1.03319
9	newly 3 weeks spec	28.	83.2	{1., 132.}	268.286	0.764029	newly 3 weeks cumul	137.	524.63	{12., 917.}	333.175	0.619716	newly 5 weeks spec	9.	51.81	{0., 77.}	556.556	1.03085	newly 5 weeks cumul	177.	634.9	{16., 997.}	308.599	0.618736	newly 50 weeks cumul	178.	1480.01	{19., 5540.}	770.545	0.769687
10	newly 3 weeks spec	9.	112.25	{7., 78.}	1158.78	0.495633	newly 3 weeks cumul	56.	746.99	{89., 485.}	1235.3	0.558243	newly 5 weeks spec	0.1	38.79	{0., 28.}	38 726.	1.41169	newly 5 weeks cumul	64.	850.92	{92., 545.}	1231.09	0.542872	newly 50 weeks cumul	67.	894.85	{93., 545.}	1237.24	0.535751
11	newly 3 weeks spec	0.1	50.09	{0., 98.}	50 042.	1.78941	newly 3 weeks cumul	7.	1138.56	{59., 4020.}	16 165.1	1.81791	newly 5 weeks spec	0.1	12.44	{0., 33.}	12 434.	1.14718	newly 5 weeks cumul	7.	1175.46	{99., 4020.}	16 692.3	1.86099	newly 49 weeks cumul	7.	1201.7	{184., 4020.}	17 067.1	1.89616
12	newly 3 weeks spec	18.	29.92	{1., 62.}	144.889	0.621829	newly 3 weeks cumul	97.	582.63	{100., 966.}	512.794	0.665743	newly 5 weeks spec	11.	3.18	{0., 9.}	96.1818	1.55052	newly 5 weeks cumul	121.	594.02	{101., 1036.}	404.364	0.594497	newly 49 weeks cumul	154.	596.54	{101., 1036.}	302.948	0.516563
13	newly 3 weeks spec	27.	143.34	{13., 235.}	456.444	0.658786	newly 3 weeks cumul	156.	1453.12	{463., 3354.}	833.141	0.77824	newly 5 weeks spec	15.	49.69	{0., 131.}	294.467	0.853693	newly 5 weeks cumul	190.	1582.6	{486., 3548.}	734.821	0.747564	newly 46 weeks cumul	207.	1684.23	{486., 3732.}	715.589	0.745459
14	newly 3 weeks spec	5.	131.9	{7., 141.}	2545.6	0.948405	newly 3 weeks cumul	40.	519.33	{38., 532.}	1208.33	0.723315	newly 5 weeks spec	0.1	93.64	{3., 93.}	93 546.	2.20666	newly 5 weeks cumul	41.	721.08	{49., 690.}	1667.07	0.817141	newly 48 weeks cumul	41.	1092.83	{64., 1690.}	2572.51	0.91632
15	newly 3 weeks spec	2.	227.29	{0., 800.}	11 292.5	1.11059	newly 3 weeks cumul	22.	1115.94	{171., 2453.}	4973.91	1.23768	newly 5 weeks spec	2.	77.15	{0., 152.}	3875.5	1.30676	newly 5 weeks cumul	26.	1329.62	{205., 3728.}	5015.46	1.20402	newly 52 weeks cumul	28.	1421.63	{223., 5054.}	4978.82	1.19314
16	newly 3 weeks spec	8.	219.05	{0., 1003.}	2710.13	1.13646	newly 3 weeks cumul	58.	1505.52	{172., 8672.}	2498.34	0.877107	newly 5 weeks spec	4.	33.94	{0., 73.}	860.	1.17134	newly 5 weeks cumul	73.	1626.12	{172., 8674.}	2130.47	0.803091	newly 48 weeks cumul	90.	1649.77	{172., 8674.}	1736.19	0.722584
17	newly 3 weeks spec	89.	74.51	{0., 207.}	135.067	1.72855	newly 3 weeks cumul	412.	1188.1	{136., 4027.}	275.714	0.50682	newly 5 weeks spec	62.	13.83	{0., 19.}	100.661	2.18701	newly 5 weeks cumul	564.	1233.23	{138., 4060.}	221.559	0.574638	newly 48 weeks cumul	769.	1242.14	{138., 4060.}	177.212	0.64434
18	newly 3 weeks spec	67.	13.45	{0., 37.}	89.5373	1.95083	newly 3 weeks cumul	293.	301.76	{18., 408.}	77.4334	0.391055	newly 5 weeks spec	37.	5.31	{0., 13.}	91.4324	1.89568	newly 5 weeks cumul	379.	316.05	{22., 438.}	73.9446	0.439669	newly 46 weeks cumul	562.	331.1	{28., 550.}	74.7509	0.55702
19	newly 3 weeks spec	35.	37.29	{0., 76.}	140.143	1.43214	newly 3 weeks cumul	159.	581.6	{23., 496.}	295.962	0.426247	newly 5 weeks spec	32.	5.69	{0., 12.}	94.0938	1.80609	newly 5 weeks cumul	221.	601.13	{25., 599.}	215.851	0.402353	newly 42 weeks cumul	292.	605.41	{31., 643.}	170.86	0.430946
20	newly 3 weeks spec	92.	64.32	{0., 118.}	105.63	1.74865	newly 3 weeks cumul	401.	1575.59	{84., 5518.}	318.8	0.520952	newly 5 weeks spec	59.	8.43	{0., 26.}	92.8983	1.95076	newly 5 weeks cumul	544.	1606.82	{96., 5518.}	224.143	0.444909	newly 42 weeks cumul	745.	1613.08	{96., 5518.}	157.332	0.408943
21	newly 3 weeks spec	36.	82.28	{3., 47.}	239.889	0.626968	newly 3 weeks cumul	108.	450.98	{61., 414.}	363.333	0.340316	newly 5 weeks spec	14.	23.59	{0., 15.}	206.214	1.00856	newly 5 weeks cumul	144.	520.43	{66., 442.}	321.84	0.37416	newly 50 weeks cumul	181.	545.57	{66., 448.}	275.133	0.408636
22	newly 3 weeks spec	13.	102.21	{0., 252.}	742.077	0.910195	newly 3 weeks cumul	94.	1746.25	{389., 5472.}	1757.71	1.03446	newly 5 weeks spec	3.	38.5	{0., 61.}	1282.67	1.09317	newly 5 weeks cumul	123.	1835.71	{507., 5497.}	1392.72	0.950502	newly 48 weeks cumul	123.	1991.47	{569., 5824.}	1519.36	0.976764
23	newly 3 weeks spec	73.	101.14	{0., 298.}	168.795	1.01485	newly 3 weeks cumul	250.	749.23	{164., 1220.}	229.66	0.262084	newly 5 weeks spec	30.	35.21	{0., 158.}	165.233	1.47743	newly 5 weeks cumul	319.	841.64	{175., 1442.}	209.129	0.292587	newly 47 weeks cumul	375.	947.31	{176., 2869.}	211.837	0.343437
24	newly 3 weeks spec	15.	171.86	{0., 68.}	1120.53	0.7812	newly 3 weeks cumul	84.	769.59	{91., 777.}	823.369	0.550898	newly 5 weeks spec	11.	55.46	{0., 19.}	553.636	1.31464	newly 5 weeks cumul	110.	939.95	{95., 801.}	764.718	0.483991	newly 48 weeks cumul	115.	978.77	{95., 807.}	762.043	0.475491
25	newly 3 weeks spec	0.1	81.29	{0., 82.}	81 304.	1.25358	newly 3 weeks cumul	2.	1766.83	{102., 8674.}	88 241.5	2.40778	newly 5 weeks spec	0.1	77.02	{0., 44.}	77 034.	1.15657	newly 5 weeks cumul	2.	1928.43	{102., 8674.}	96 321.5	2.4689	newly 51 weeks cumul	2.	2259.07	{108., 8749.}	112 854.	2.52953
26	newly 3 weeks spec	24.	260.59	{3., 1533.}	1039.04	0.717184	newly 3 weeks cumul	102.	2079.74	{158., 6803.}	1950.22	0.979989	newly 5 weeks spec	5.	36.55	{0., 140.}	752.2	1.2164	newly 5 weeks cumul	124.	2222.43	{159., 7371.}	1704.02	0.923764	newly 50 weeks cumul	128.	2242.63	{159., 7496.}	1663.87	0.913903
27	newly 3 weeks spec	35.	45.51	{0., 123.}	144.429	1.60175	newly 3 weeks cumul	192.	954.44	{143., 650.}	414.104	0.402775	newly 5 weeks spec	19.	57.7	{0., 164.}	321.368	1.53507	newly 5 weeks cumul	236.	1062.65	{168., 1408.}	368.097	0.412213	newly 51 weeks cumul	284.	1820.37	{182., 8235.}	563.398	0.508901
28	newly 3 weeks spec	7.	128.64	{0., 479.}	1801.43	1.14049	newly 3 weeks cumul	31.	628.02	{53., 1584.}	1934.52	0.924946	newly 5 weeks spec	4.	83.31	{0., 313.}	2071.75	1.30661	newly 5 weeks cumul	37.	811.	{71., 2566.}	2100.16	0.937405	newly 50 weeks cumul	43.	1347.99	{93., 4694.}	3043.51	1.0122
29	newly 3 weeks spec	0.1	245.43	{41., 318.}	245 330.	3.0644	newly 3 weeks cumul	12.	1483.74	{394., 1865.}	12 264.5	1.87897	newly 5 weeks spec	0.1	43.51	{3., 82.}	43 416.	2.28907	newly 5 weeks cumul	12.	1630.6	{459., 2121.}	13 488.3	1.93053	newly 52 weeks cumul	12.	1683.43	{488., 2192.}	13 928.6	1.95692
30	newly 3 weeks spec	0.1	87.42	{1., 166.}	87 330.	2.39106	newly 3 weeks cumul	4.	872.03	{113., 934.}	21 700.8	1.93881	newly 5 weeks spec	0.1	31.21	{0., 68.}	31 180.	1.53344	newly 5 weeks cumul	4.	952.84	{113., 1026.}	23 721.	1.98532	newly 52 weeks cumul	4.	1030.21	{113., 1083.}	25 655.3	2.01309
31	newly 3 weeks spec	16.	22.06	{0., 47.}	103.625	0.833179	newly 3 weeks cumul	78.	1061.24	{158., 6217.}	1260.56	0.622461	newly 5 weeks spec	25.	7.06	{0., 18.}	76.08	1.24363	newly 5 weeks cumul	122.	1081.22	{172., 6217.}	786.246	0.465978	newly 49 weeks cumul	141.	1092.72	{176., 6217.}	674.979	0.420455
32	newly 3 weeks spec	66.	331.37	{105., 627.}	405.045	0.526465	newly 3 weeks cumul	192.	1765.81	{851., 2421.}	821.974	0.815734	newly 5 weeks spec	25.	127.22	{20., 271.}	418.96	0.498384	newly 5 weeks cumul	260.	2097.54	{1048., 2990.}	708.608	0.757062	newly 42 weeks cumul	338.	2352.08	{1091., 3283.}	596.503	0.689296
33	newly 3 weeks spec	53.	29.5	{1., 60.}	92.7925	0.933346	newly 3 weeks cumul	200.	362.38	{33., 433.}	109.77	0.265101	newly 5 weeks spec	33.	6.38	{0., 21.}	90.2424	1.76949	newly 5 weeks cumul	277.	381.41	{43., 467.}	94.8123	0.316641	newly 42 weeks cumul	389.	390.1	{48., 487.}	81.0386	0.376827
34	newly 3 weeks spec	62.	121.32	{8., 337.}	188.484	0.57136	newly 3 weeks cumul	247.	1551.27	{370., 4869.}	528.045	0.502891	newly 5 weeks spec	32.	13.44	{0., 32.}	97.25	1.35226	newly 5 weeks cumul	321.	1605.64	{372., 5014.}	400.199	0.400064	newly 42 weeks cumul	401.	1612.37	{372., 5015.}	305.309	0.319363
35	newly 3 weeks spec	19.	39.16	{0., 87.}	218.421	0.868654	newly 3 weeks cumul	121.	724.09	{90., 1917.}	510.223	0.555962	newly 5 weeks spec	16.	4.53	{0., 14.}	98.6875	1.76034	newly 5 weeks cumul	158.	741.13	{92., 1919.}	383.677	0.474048	newly 49 weeks cumul	183.	744.44	{92., 1919.}	322.907	0.42797
36	newly 3 weeks spec	68.	35.83	{0., 93.}	73.0147	1.74004	newly 3 weeks cumul	286.	1660.49	{315., 7476.}	481.29	0.394996	newly 5 weeks spec	63.	18.92	{0., 54.}	77.6825	1.77164	newly 5 weeks cumul	404.	1704.89	{349., 7476.}	326.918	0.30817	newly 45 weeks cumul	496.	1760.84	{367., 7476.}	265.315	0.287297
37	newly 3 weeks spec	40.	75.99	{0., 231.}	218.475	1.44225	newly 3 weeks cumul	178.	1113.96	{140., 3857.}	535.652	0.364091	newly 5 weeks spec	13.	28.79	{0., 47.}	273.154	1.53605	newly 5 weeks cumul	219.	1177.67	{152., 3857.}	453.995	0.338866	newly 48 weeks cumul	249.	1280.36	{155., 4646.}	438.378	0.351619
38	newly 3 weeks spec	60.	108.25	{0., 344.}	218.117	1.62566	newly 3 weeks cumul	237.	963.58	{143., 2001.}	343.755	0.338306	newly 5 weeks spec	33.	45.44	{0., 127.}	199.152	1.95435	newly 5 weeks cumul	316.	1071.75	{144., 3435.}	299.13	0.405193	newly 48 weeks cumul	375.	1184.38	{144., 4343.}	288.491	0.455972
39	newly 3 weeks spec	66.	129.23	{10., 161.}	182.955	0.504319	newly 3 weeks cumul	292.	487.45	{112., 830.}	127.887	0.386295	newly 5 weeks spec	58.	116.23	{1., 40.}	251.638	1.06174	newly 5 weeks cumul	396.	743.85	{120., 947.}	158.538	0.422462	newly 44 weeks cumul	641.	929.6	{121., 1656.}	133.934	0.473078
40	newly 3 weeks spec	57.	197.46	{0., 724.}	391.123	1.90625	newly 3 weeks cumul	264.	1640.24	{221., 6106.}	533.5	0.465614	newly 5 weeks spec	47.	74.92	{0., 50.}	225.489	2.18287	newly 5 weeks cumul	347.	1835.48	{222., 7365.}	453.222	0.442739	newly 44 weeks cumul	489.	1948.76	{222., 8154.}	346.483	0.45807
41	newly 3 weeks spec	16.	353.42	{2., 1170.}	2142.88	0.98582	newly 3 weeks cumul	51.	2237.82	{64., 8698.}	4292.12	1.12045	newly 5 weeks spec	0.1	75.3	{0., 132.}	75 240.	1.96153	newly 5 weeks cumul	55.	2467.3	{64., 9321.}	4391.05	1.14655	newly 52 weeks cumul	59.	2632.62	{64., 9371.}	4367.86	1.15875
42	newly 3 weeks spec	25.	109.17	{0., 158.}	378.76	0.649457	newly 3 weeks cumul	108.	1353.59	{64., 1346.}	1167.05	0.827588	newly 5 weeks spec	2.	36.17	{0., 80.}	1764.5	0.892537	newly 5 weeks cumul	125.	1446.94	{83., 2355.}	1069.55	0.79835	newly 43 weeks cumul	125.	1617.5	{100., 7070.}	1203.34	0.82239
43	newly 3 weeks spec	9.	28.08	{0., 87.}	350.	1.60191	newly 3 weeks cumul	69.	1332.97	{152., 7168.}	1831.84	0.924518	newly 5 weeks spec	6.	28.52	{0., 52.}	514.	1.4886	newly 5 weeks cumul	83.	1391.04	{217., 7168.}	1575.95	0.895113	newly 51 weeks cumul	83.	1662.8	{255., 7691.}	1903.37	0.96985
44	newly 3 weeks spec	52.	253.28	{32., 410.}	406.462	0.429398	newly 3 weeks cumul	260.	1274.49	{146., 3134.}	405.742	0.445145	newly 5 weeks spec	27.	79.82	{10., 133.}	237.704	0.391742	newly 5 weeks cumul	320.	1496.97	{182., 3391.}	385.272	0.440166	newly 45 weeks cumul	421.	1634.66	{228., 3460.}	313.387	0.416783
45	newly 3 weeks spec	23.	310.55	{23., 1082.}	1254.39	0.541471	newly 3 weeks cumul	107.	1054.36	{228., 3097.}	885.383	0.670978	newly 5 weeks spec	11.	60.19	{2., 225.}	495.909	0.605191	newly 5 weeks cumul	130.	1263.29	{262., 3943.}	871.762	0.645949	newly 48 weeks cumul	143.	1324.92	{289., 4152.}	826.517	0.637145
46	newly 3 weeks spec	21.	152.99	{4., 472.}	669.952	0.670232	newly 3 weeks cumul	150.	2053.22	{430., 5700.}	1270.61	0.849879	newly 5 weeks spec	19.	31.56	{0., 83.}	190.737	1.33045	newly 5 weeks cumul	189.	2152.85	{450., 5957.}	1041.53	0.773571	newly 47 weeks cumul	195.	2183.29	{460., 5957.}	1022.2	0.766652
47	newly 3 weeks spec	36.	117.29	{0., 327.}	322.472	1.12881	newly 3 weeks cumul	193.	1351.65	{183., 2098.}	610.544	0.459305	newly 5 weeks spec	17.	42.43	{0., 157.}	290.294	1.29862	newly 5 weeks cumul	233.	1466.33	{183., 3362.}	542.416	0.430054	newly 48 weeks cumul	246.	1515.92	{183., 4545.}	530.943	0.425822
48	newly 3 weeks spec	58.	189.78	{7., 279.}	283.	0.555117	newly 3 weeks cumul	260.	1628.28	{161., 4414.}	543.662	0.590234	newly 5 weeks spec	33.	127.79	{0., 285.}	393.606	1.37385	newly 5 weeks cumul	358.	1904.99	{186., 5336.}	452.377	0.556102	newly 45 weeks cumul	432.	2426.61	{225., 8117.}	480.637	0.561305
49	newly 3 weeks spec	33.	84.25	{0., 43.}	255.788	0.86995	newly 3 weeks cumul	141.	548.85	{12., 777.}	338.745	0.401247	newly 5 weeks spec	29.	44.08	{0., 25.}	194.828	1.29298	newly 5 weeks cumul	202.	648.31	{13., 1660.}	289.965	0.461338	newly 47 weeks cumul	230.	963.62	{13., 5355.}	388.478	0.497978
50	newly 3 weeks spec	0.1	133.25	{0., 219.}	133 194.	2.01445	newly 3 weeks cumul	33.	935.08	{10., 2511.}	2763.39	0.87901	newly 5 weeks spec	0.1	81.71	{0., 36.}	81 680.	1.46852	newly 5 weeks cumul	33.	1115.03	{10., 4626.}	3306.27	0.936289	newly 52 weeks cumul	33.	1346.6	{10., 5519.}	4006.91	0.994305
